# Analysis of diagnosis delay among migrant pulmonary tuberculosis patients in Guangzhou from 2014 to 2022

**DOI:** 10.3389/fpubh.2025.1399688

**Published:** 2025-05-14

**Authors:** Yajuan Feng, Keng Lai, Jieying Yang, Yu Lei, Guifeng Wu, Yuhua Du, Weiyun He

**Affiliations:** State Key Laboratory of Respiratory Disease, Guangzhou Key Laboratory of Tuberculosis Research, Department of Tuberculosis Control and Management, Guangzhou Chest Hospital, Institute of Tuberculosis, Guangzhou Medical University, Guangzhou, Guangdong, China

**Keywords:** delay, tuberculosis, migrants, risk factor, bacteriological

## Abstract

**Background:**

Delays in diagnosing pulmonary tuberculosis (PTB) lead to more severe disease progression and sustained transmission within communities. This study aimed to assess trends and identify risk factors associated with PTB diagnosis delays, especially among migrants, in Guangzhou from 2014 to 2022.

**Methods:**

Demographic and clinical information of PTB patients from 2014 to 2022 in Guangzhou were extracted from the PTB Information Management System (TBIMS). Diagnosis delays were categorized as patient delay (>30 days from symptoms onset to first care-seeking) and hospital delay (>14 days from first care-seeking to TB diagnosis). Multivariable logistic regression was used to analyze the risk factors for these delays.

**Results:**

A total of 35,722 migrant PTB patients were registered in Guangzhou during the study period, exhibiting distinct characteristics compared to local residents (*n* = 44,155). The bacteriological positivity rate among migrants was significantly lower than that of the local residents (47.68% vs. 52.41%, *p* < 0.001). Overall, 44.39% patients experienced a diagnosis delay, comprising 27.90% with patient delay and 29.09% with hospital delay, with both showing a gradually declining trend over time. Multivariate logistic regression analysis revealed risk factors for diagnosis delay including female sex, older age, ethnic minority status, migrant status and re-treated patients, which were similar to patient delay. Risk factors for hospital delay included, passive case finding, and re-treated patients. Notably, sputum smear-positivity was positively associated with both the diagnostic delay (aOR 1.279, 95% CI: 1.224–1.335) and patient delay (aOR 1.642, 95% CI: 1.563–1.724), but reduced the risk of hospital delay (aOR 0.906, 95% CI: 0.866–0.950).

**Conclusion:**

Public health strategies should prioritize improving bacteriological detection rates among migrants, with targeted efforts for high-risk groups such as females, the older adult, and ethnic minorities.

## 1 Introduction

Pulmonary tuberculosis (PTB), an infectious disease caused by *Mycobacterium tuberculosis* (Mtb), is the second leading cause of death from a single infectious disease worldwide. According to the 2023 global tuberculosis report, the mortality rate of PTB in 2022 was second only to that of coronavirus disease (COVID-19), almost twice that of HIV/AIDS (1, 2). This underscores the urgency of effectively preventing and controlling PTB.

Generally, PTB is a preventable and usually curable disease (3). A critical component of PTB prevention is early diagnosis. However, early symptoms of PTB are often non-specific, such as coughing and sputum production, which are prevalent in most cases, particularly in the older adult with underlying diseases (4, 5), immunocompromised individuals and smokers (6). This can lead to delayed interaction with health services, resulting in a delayed suspicion of PTB (7–9). However, with patients experiencing delays in seeking medical help and confirmation of diagnosis, this not only affect individual health, disease prognosis or even led to death, but also significantly increase the possibility of transmission, particularly among close household contacts (10, 11).

Since PTB is a social disease that requires social, economic, and environmental interventions (12), determinants such as poverty, malnutrition, HIV infection, smoking and diabetes could affect the number of PTB infections and the incidence of the disease (13). PTB hits the hardest among the poor and vulnerable populations, especially migrants (14). In fact, migrants often face significant barriers to subsidized housing, social security, healthcare and insurance in urban centers. They are more likely to share crowded living conditions, have a lower socioeconomic status and have a lower level of formal education. These factors often led to a greater burden of disease for them.

As one of the most important economic centers in southern China, Guangzhou attracts a large number of migrants from other provinces of China and overseas with its favorable geographical location, developed manufacturing and service industries, among other job opportunities. According to the data from the Guangzhou Bureau of Statistics (15), by the end of 2022, the registered population of Guangzhou is 10.3491 million, and the population of migrants is 8.385 million, accounting for approximately half of the total population. This indicates that Guangzhou can be regarded as a typical migrant city. However, the PTB preferential policies in Guangzhou only benefit registered local residents, excluding migrants. Currently, the situation of PTB among migrants is severe in Guangzhou. Delays in migrant PTB patients can lead to difficulties in prevention and control, causing the transmission of the epidemic. In order to draw the attention of decision-making departments and to tilt more funding into policies that benefit migrants, our article focused on analyzing the trend of delays and risk factors among migrant PTB patients in Guangzhou from 2014 to 2022. The findings could serve as a theoretical basis for formulating policies to prevent and control PTB among migrant patients.

## 2 Methods

### 2.1 Study setting and data collection

Data regarding PTB patients from 2014 to 2022 in Guangzhou was extracted from the PTB Information Management System (TBIMS), a standardized PTB patient registration system established by the Chinese Center for Disease Control and Prevention (CDC) for reporting and managing PTB patients. All healthcare providers were required to report suspected or laboratory-confirmed PTB cases within 24 h and refer these cases to designated PTB hospitals for diagnosis and treatment. Data regarding PTB patients was collected and entered into TBIMS by trained physicians at designated hospitals and checked daily by dedicated public health physicians (16, 17).

We collected the demographic and clinical characteristics of the patients, including gender (male, female), age (<20 years, 21–30 years, 31–40 years, 41–50 years, 51–60 years, ≥61 years), ethnicity (Han, minority), census register (local resident, migrant), occupation (unemployment, worker, official staff, waiter and retired staff), case finding (active, passive), treatment history (newly treated, re-treated), bacteriological results (negative, positive) and first sputum smear status (negative, positive). Patients with simple TB pleurisy or extrapulmonary TB and those with missing key information were excluded from the analysis.

These migrants were defined as those without a Guangzhou household registration status through the Chinese Hukou system. The remaining classifications were determined according to the TBIMS system’s categorization. In the occupational classification, “waiter” referred to service industries such as catering and customer service. “worker” referred to individuals engaged in manual labor or production activities, including those in manufacturing and construction.

### 2.2 Critical definitions

Active pulmonary TB patients were diagnosed based on the National Guidelines for PTB Diagnosis in both inpatients and outpatients (18, 19). Suspected patients provided three sputum specimens for bacteriological confirmation. And patients with positive bacterial cultures were confirmed as such if at least one of the following tests was positive: sputum smear, culture, or molecular testing.

The diagnosis delay, defined as the total time interval between the symptom onset and final diagnosis of TB, encompassing two components: patient delay (exceeding 30 days between symptom onset and initial healthcare-seeking) and hospital delay (exceeding 14 days between first healthcare-seeking to the confirmed diagnosis of TB). A case was considered to have diagnosis delay if either patient delay or hospital delay was present (11, 17, 20, 21).

### 2.3 Statistical analysis

The data was collected and cleaned in Excel. Continuous variables were presented as mean (standard deviation, SD) or medians (interquartile range, IQR), and categorical variables were presented as n (percent). Differences in categorical variables between groups were compared using a chi-square test to screen for the related factors of patient and hospital delays. Variables with a *p*-value <0.05 were included in the multivariable logistic regression model to estimate the adjusted odds ratios (aOR) with the 95% confidence intervals (CI) of the independent risk factors. The difference was considered statistically significant when *p* < 0.05. All the statistical analyses were performed in SPSS 24.0 and R (v4.2.2).

## 3 Results

### 3.1 Study population

**Figure 1:**
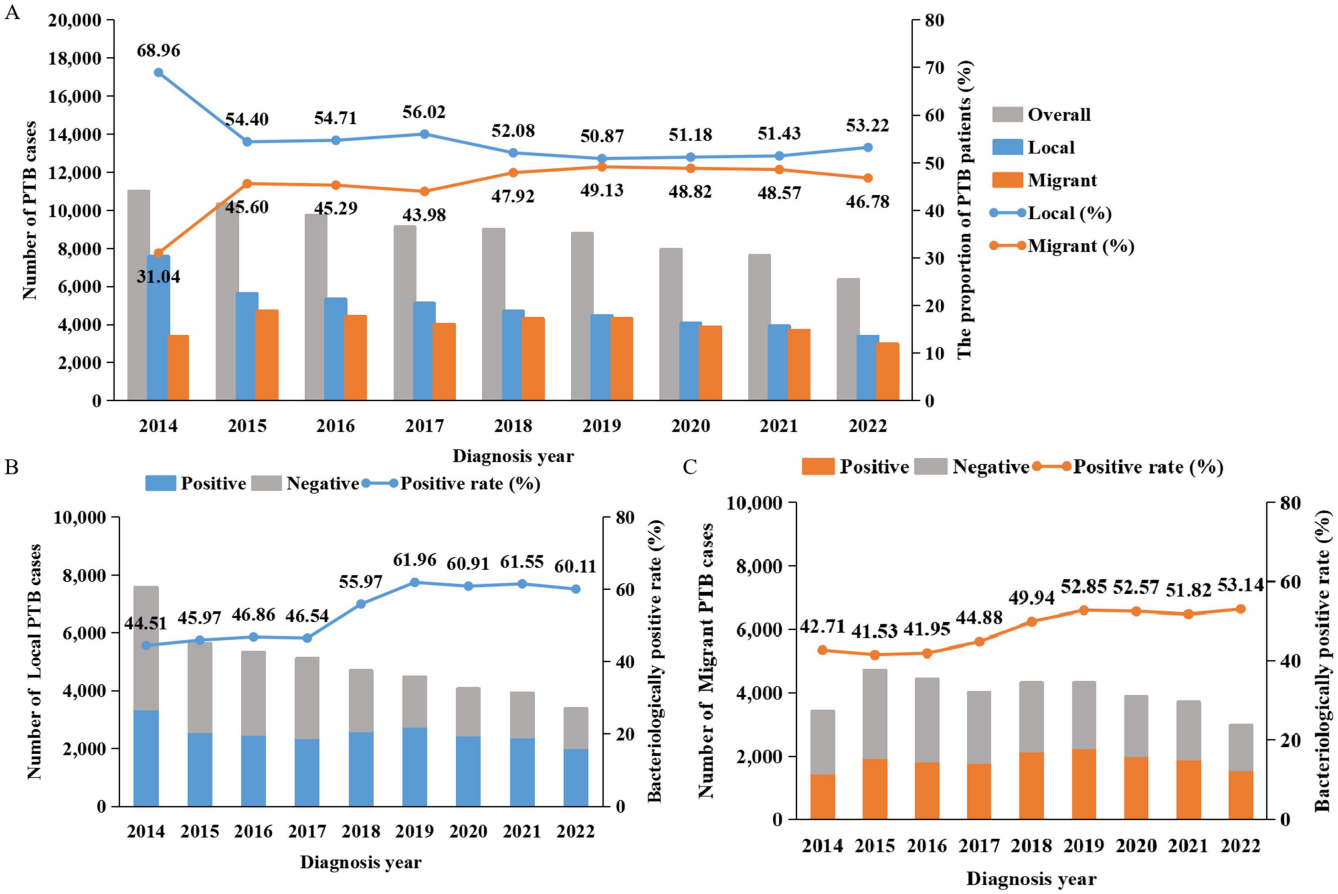
Numbers, proportions and bacteriological results of PTB patients in Guangzhou from 2014 to 2022. **(A)** The bar charts show the number of PTB patients, the line graph shows the proportions of local residents and migrants among PTB patients. Gray represents the total number, blue represents local residents, and orange represents migrants. **(B,C)** The cumulative bar charts display the bacteriological results of local and migrant PTB patients. The line graphs represent the bacteriological positive rates. Gray indicates the number of negative cases, blue represents local residents, and orange represents migrants.

From 2014 to 2022, a total of 79,877 active TB patients were registered in Guangzhou, with the number of registered patients decreasing each year, from 10,989 to 6,355 patients. Among them, 35,722 cases (44.72%) were migrant PTB patients. The proportion of migrant PTB patients went from 31.04% in 2014 to 46.78% in 2022 (Figure 1), this proportion increased year by year, but in the last 5 years stabilized.

Migrants differed significantly from local residents in various characteristics, including a higher percentage of females (34.14% vs. 30.69%) and ethnic minority patients (3.49% vs. 0.57%). The age groups of the migrants were mainly 21–30 years old (36.10%) and 31–40 years old (20.68%), while among the local residents, the age group with the largest proportion was those over 61 years old (30.38%). Additionally, 95.08% of migrants were newly treated patients, with occupations mainly including unemployed (60.46%), waiter (12.14%), worker (21.04%). The proportion of active patient case finding (50.50%) among migrants was lower than that (56.56%) among the local residents (*χ*^2^ = 291.19, *p* < 0.001). Moreover, 11,675 migrant cases (32.68%) tested positive for their first sputum smear (Table 1).

**Table 1:** Basic characteristics of the PTB patients in Guangzhou from 2014 to 2022, stratified by the census register status.

Characters	Total (*n* = 79,877)	Local resident (*n* = 44,155)	Migrant (*n* = 35,722)	*χ* ^2^	*p*-value
Gender				107.40	<0.001
Male	54,130 (67.77)	30,603 (69.31)	23,527 (65.86)		
Female	25,747 (32.23)	13,552 (30.69)	12,195 (34.14)		
Age				9,878.51	<0.001
≦20	5,636 (7.06)	3,050 (6.91)	2,586 (7.24)		
21–30	20,302 (25.42)	7,405 (16.77)	12,897 (36.10)		
31–40	13,030 (16.31)	5,644 (12.78)	7,386 (20.68)		
41–50	12,540 (15.70)	6,284 (14.23)	6,256 (17.51)		
51–60	12,414 (15.54)	8,357 (18.93)	4,057 (11.36)		
≧61	15,955 (19.97)	13,415 (30.38)	2,540 (7.11)		
Ethnicity				913.27	<0.001
Han	78,379 (98.12)	43,903 (99.43)	34,476 (96.51)		
Minority	1,498 (1.88)	252 (0.57)	1,246 (3.49)		
Occupation				6,110.06	<0.001
Unemployment	42,699 (53.46)	21,102 (47.79)	21,597 (60.46)		
Worker	17,152 (21.47)	9,635 (21.82)	7,517 (21.04)		
Official staff	3,734 (4.67)	2,350 (5.32)	1,384 (3.87)		
Waiter	7,040 (8.81)	2,705 (6.13)	4,335 (12.14)		
Retired staff	9,252 (11.58)	8,363 (18.94)	889 (2.49)		
Case finding				291.19	<0.001
Active	43,014 (53.85)	24,973 (56.56)	18,041 (50.50)		
Passive	36,863 (46.15)	19,182 (43.44)	17,681 (49.50)		
Treatment history				341.80	<0.001
Newly treated	74,489 (93.25)	40,525 (91.78)	33,964 (95.08)		
Re-treated	5,388 (6.75)	3,630 (8.22)	1,758 (4.92)		
First sputum smear status				205.51	<0.001
Negative	51,617 (64.62)	27,570 (62.44)	24,047 (67.32)		
Positive	28,260 (35.38)	16,585 (37.56)	11,675 (32.68)		
Bacteriological results				176.42	<0.001
Negative	39,701 (49.70)	21,013 (47.59)	18,688 (52.32)		
Positive	40,176 (50.30)	23,142 (52.41)	17,034 (47.68)		
Patient delay^a^	12 (1,35)	12 (1,34)	11 (1,36)	3.92	0.048
≤30 days	57,594 (72.10)	31,962 (72.39)	25,632 (71.75)		
>30 days	22,283 (27.90)	12,193 (27.61)	10,090 (28.25)		
Hospital delay^a^	6 (1,17)	6 (1,17)	6 (2,17)	5.39	0.020
≤14 days	56,640 (70.91)	31,458 (71.24)	25,182 (70.49)		
>14 days	23,237 (29.09)	12,697 (28.76)	10,540 (29.51)		
Diagnosis delay^a^	26 (12,60)	26 (12.59)	27 (12,60)	2.91	0.088
No	44,422 (55.61)	24,675 (55.88)	19,747 (55.28)		
Yes	35,455 (44.39)	19,480 (44.12)	15,975 (44.72)		

Data are presented as the n (percentages), Statistical significance between local residents and migrants were tested by the chi-square.

^a^The delay time of the patient was represented by the median (quartile), and whether there was a delay was represented by n (percentage). The difference in whether there was a delay between local residents and migrants was compared using chi-square values.

### 3.2 The trend of bacteriological positivity rate

During the study period, 50.30% (40,176/79,877) of patients were diagnosed with bacteriologically confirmed pulmonary tuberculosis, and this proportion showed a gradual upward trend. The bacteriological positivity rate of PTB patients among local residents gradually increased from 44.51% (3,373/7,578) in 2014 to 46.54% (2,378/5,110) in 2017, then experienced a sharp rise to 55.97% (2,625/4,690) in 2018 and continued to rise to 61.96% (2,767/4,466) in 2019. Thereafter, it slightly decreased to 60.11% (2,033/3,382) in 2022. The bacteriological positivity rate of PTB patients among migrants showed a slow upward trend, rising from 42.7% (1,457/3,411) in 2014 to 53.14% (1,580/2,973) in 2022. The bacteriological positivity rate of migrant PTB patients was lower than that of local residents, and this difference was statistically significant (*χ*^2^ = 176.42, *p* < 0.001) (Table 1). A notable increase was observed in 2018, primarily attributed to the promotion of molecular testing techniques.

### 3.3 The trend of diagnosis delays

**Figure 2:**
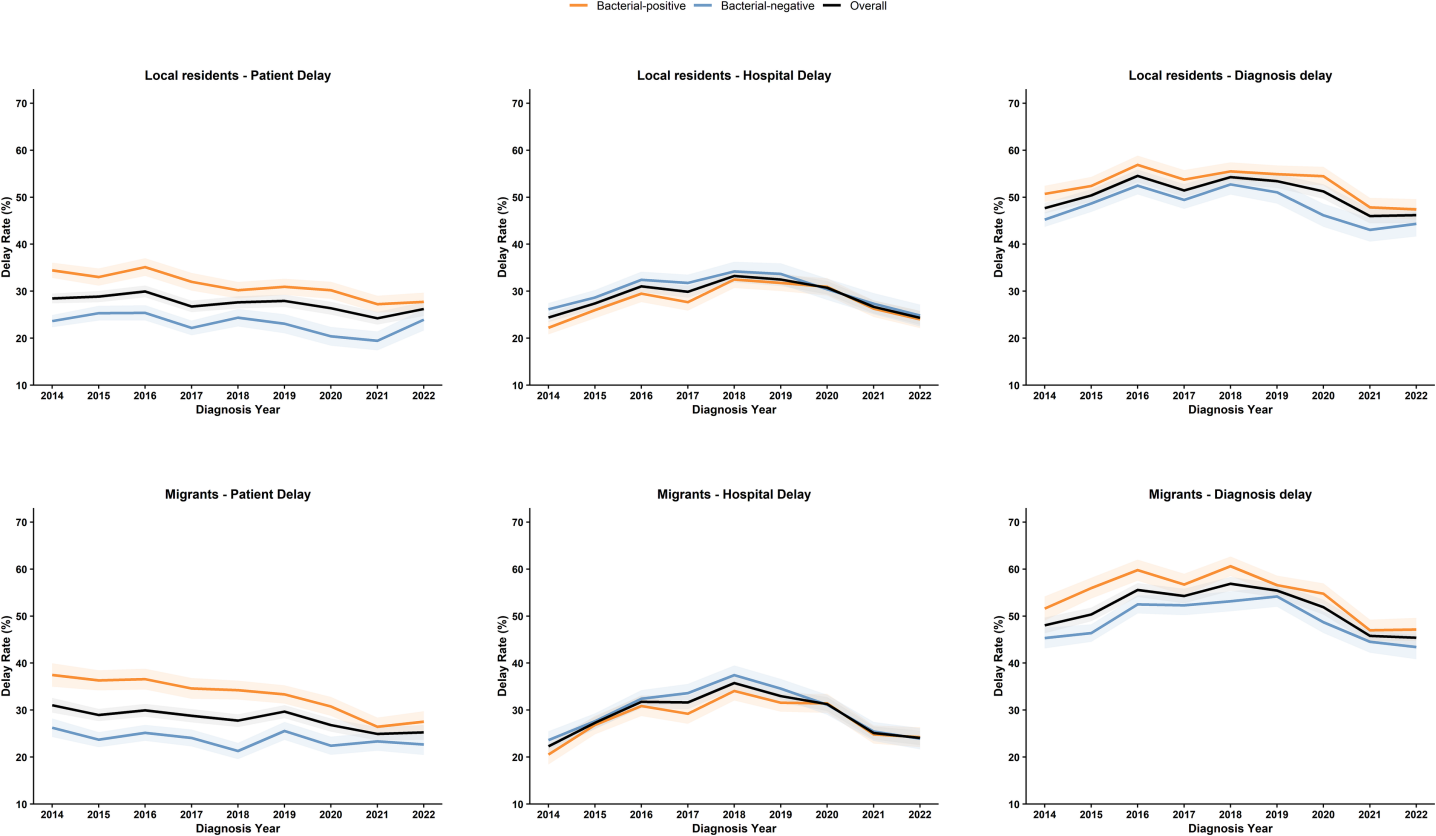
Trends in delays among local and migrant PTB patients in Guangzhou from 2014 to 2022. The figure illustrates the trends regarding patient delay, hospital delay, and diagnosis delay among PTB patients from 2014 to 2022. We conduct stratified analyses for local residents and migrants. The shaded areas denote the 95% confidence intervals of the annual estimates. Orange indicates bacteriologically positive cases, blue represents bacteriologically negative cases, and black signifies the overall situation.

To determine the trends in diagnostic delay, including patient delay and hospital delay, we analyzed the annual delay rates from 2014 to 2022 (Figure 2). Overall, the diagnostic delay rate rose from 47.78% in 2014 to a peak of 55.52% in 2018. Although it decreased by 2022, it remained at a high level of 45.82%, with a statistically significant trend (*χ*^2^ = 14.24, *p* < 0.001). The patient delay rate showed a downward trend, decreasing from 29.26% in 2014 to 25.77% in 2022, with a statistically significant trend (*χ*^2^ = 8.70, *p* < 0.001). Conversely, the overall hospital delay rate increased initially, peaking at 34.45% in 2018 before gradually declining to 24.22% in 2022, with a statistically significant trend (*χ*^2^ = 70.98, *p* < 0.001).

For migrant patients, the median diagnostic delay was 27 days (IQR 12–60 days), with the median patient delay being 11 days (IQR 1–36 days) and the median hospital delay being 6 days (IQR 2–17 days). Diagnostic delay occurred in 18,540 (51.90%) migrant patients, compared with 22,356 (50.63%) local patients, with a statistically significant difference (*χ*^2^ = 12.75, *p* < 0.001) (Table 1). A total of 28.25% (10,090/35,722) of migrants experienced patient delay, a proportion higher than that of local residents (27.61%, 12,193/44,155), with a statistically significant difference (*χ*^2^ = 3.92, *p* = 0.048). Additionally, 29.51% (10,540/35,722) of migrants had hospital delay, higher than local residents (28.76%, 12,697/44,155), with a statistically significant difference (*χ*^2^ = 5.39, *p* = 0.020).

### 3.4 Risk factors of diagnosis delays

In the logistic regression model of diagnostic delay, significant differences between delayed and non-delayed patients were observed in age, ethnicity, household registration, occupation, case-finding method, treatment history, initial sputum smear positivity, and bacteriological results (Supplementary Table 3). The multivariate model identified independent risk factors for diagnostic delay as female sex (aOR 1.057, 95% CI: 1.025–1.089, *p* < 0.001); older age groups (41–50 years: aOR 1.094, 95% CI: 1.026–1.167; 51–60 years: aOR 1.094, 95% CI: 1.026–1.167; ≥61 years: aOR 1.106, 95% CI: 1.033–1.185); migrant population (aOR 1.074, 95% CI: 1.042–1.107, *p* < 0.001); re-treated patients (aOR 1.135, 95% CI: 1.072–1.201, *p* < 0.001); and sputum smear-positive patients (aOR 1.279, 95% CI: 1.224–1.335, *p* < 0.001). In contrast, waiter (aOR 0.85, 95% CI: 0.808–0.895, *p* < 0.001), retired staff (aOR 0.939, 95% CI: 0.888–0.994, *p* = 0.030) had a lower risk of diagnostic delay. Additionally, minority (aOR 1.049, 95% CI: 0.945–1.164, *p* = 0.369) worker (aOR 1.017, 95% CI: 0.98–1.055, *p* = 0.369), official staff (aOR 0.939, 95% CI: 0.888–0.994, *p* = 0.081) and bacteriological positivity (aOR 1.013, 95% CI: 0.972–1.056, *p* = 0.530) showed no significant association with diagnostic delay in the model (Table 2).

**Table 2:** Risk factors for patient, hospital and diagnosis delays identified by multivariable logistic regression.

Characters	Diagnosis delay		Patient delay		Hospital delay	
	aOR (95%CI)	*p*-value	aOR (95%CI)	*p*-value	aOR (95%CI)	*p*-value
Sex: female (vs. male)	**1.057 (1.025, 1.089)**	**<0.001**	**1.111 (1.073, 1.149)**	**<0.001**	0.984 (0.954, 1.018)	0.358
Age: (vs. ≦20 years)						
21–30 years	1 (0.942, 1.063)	0.989	**1.094 (1.019, 1.174)**	**0.013**	**0.933 (0.873, 0.996)**	**0.037**
31–40 years	1.051 (0.986, 1.12)	0.127	**1.249 (1.16, 1.345)**	**<0.001**	**0.897 (0.837, 0.961)**	**0.002**
41–50 years	**1.094 (1.026, 1.167)**	**0.006**	**1.35 (1.253, 1.454)**	**<0.001**	**0.902 (0.842, 0.967)**	**0.004**
51–60 years	**1.094 (1.026, 1.167)**	**0.006**	**1.405 (1.304, 1.514)**	**<0.001**	**0.871 (0.811, 0.935)**	**<0.001**
≧61 years	**1.106 (1.033, 1.185)**	**0.004**	**1.425 (1.317, 1.541)**	**<0.001**	**0.883 (0.813, 0.951)**	**0.001**
Ethic: minority (vs. Han)	1.049 (0.945, 1.164)	0.369	**1.197 (1.071, 1.338)**	**0.002**	0.905 (0.805, 1.015)	0.088
Census register: migrant (vs. local)	**1.074 (1.042, 1.107)**	**<0.001**	**1.114 (1.077, 1.153)**	**<0.001**	1.005 (0.975, 1.039)	0.770
Occupation (vs. unemployment)						
Worker	1.017 (0.980, 1.055)	0.369	0.99 (0.951, 1.031)	0.631	1.018 (0.978, 1.059)	0.395
Official staff	0.941 (0.879, 1.007)	0.081	**0.886 (0.819, 0.959)**	**0.003**	1.006 (0.936, 1.085)	0.868
Waiter	**0.85 (0.808, 0.895)**	**<0.001**	**0.732 (0.688, 0.778)**	**<0.001**	1.037 (0.987, 1.096)	0.209
Retired staff	**0.939 (0.888, 0.994)**	**0.030**	**0.881 (0.828, 0.938)**	**<0.001**	1.005 (0.945, 1.07)	0.875
Case finding: active (vs. passive)	**0.773 (0.751, 0.796)**	**<0.001**	**0.935 (0.905, 0.965)**	**<0.001**	**0.76 (0.736, 0.784)**	**<0.001**
History: re-treated (vs. newly treated)	**1.135 (1.072, 1.201)**	**<0.001**	**1.1 (1.035, 1.169)**	**0.002**	**1.08 (0.018, 1.15)**	**0.015**
Smear-Positivity	**1.279 (1.224, 1.335)**	**<0.001**	**1.642 (1.563, 1.724)**	**<0.001**	**0.906 (0.866, 0.95)**	**<0.001**
Bacteria Positivity	1.013 (0.972, 1.056)	0.530	1.045 (0.995, 1.096)	0.076	0.981 (0.931, 1.026)	0.402

aOR, adjusted Odds Ratio; CI, Confidential Interval.The bolded items indicate statistically significant results (*P* < 0.05).

Given that the causes of patient and hospital delays may differ, we compared the demographic and clinical characteristics of patients with and without these delays. For patient delay, significant differences were observed between delayed and non-delayed patients in age, ethnicity, household registration, occupation, case-finding, treatment history, initial sputum smear positivity, and bacteriological results (Supplementary Table 1). In the multivariate logistic regression model, independent risk factors for patient delay included female sex (aOR 1.111, 95% CI: 1.073–1.149), older age groups (21–30 years: aOR 1.094, 95% CI: 1.019–1.174; 31–40 years: aOR 1.249, 95% CI: 1.160–1.345; 41–50 years: aOR 1.350, 95% CI: 1.253–1.454; 51–60 years: aOR 1.405, 95% CI: 1.304–1.514; ≥61 years: aOR 1.425, 95% CI: 1.317–1.541), ethnic minority status (aOR 1.197, 95% CI: 1.071–1.338), migrant population (aOR 1.114, 95% CI: 1.077–1.153), re-treated patients (aOR 1.100, 95% CI: 1.035–1.169), and sputum smear-positive patients (aOR 1.642, 95% CI: 1.563–1.724). Conversely, official staff (aOR 0.881, 95% CI: 0.828–0.938) and patients identified through active case-finding (aOR 0.935, 95% CI: 0.905–0.965) had a lower risk of patient delay (Table 2).

For hospital delay, significant differences between delayed and non-delayed patients were found in age, household registration, occupation, case-finding, initial sputum smear positivity, and bacteriological results (Supplementary Table 2). Multivariate analysis showed that re-treated patients (aOR 1.080, 95% CI: 1.018–1.150, *p* = 0.015) had a higher risk of hospital delay, while active case finding (aOR 0.760, 95% CI: 0.736–0.784, *p* < 0.001) and sputum smear-positive patients (aOR 0.906, 95% CI: 0.866–0.950, *p* < 0.001) had a lower risk. Compared with the reference group (0–20 years), individuals aged 21 years and older had a lower risk of hospital delay. Female sex, ethnic minority status, migrant population, occupation types and bacteriological positivity were not significantly associated with hospital delay in this model (Table 2).

### 3.5 Subgroup analysis in local and migrant PTB patients

Through subgroup multivariate logistic regression analyses with consistent covariate adjustment, we compared aORs for delays between migrant and local populations.

For diagnostic delay, in the local population, risk factors include females (aOR 1.055, 95% CI 1.012–1.1), those aged 31–40 (aOR 1.12, 95% CI 1.024–1.226), 41–50 (aOR 1.144, 95% CI 1.047–1.251), 51–60 (aOR 1.165, 95% CI 1.069–1.269), 61 years and above (aOR 1.156, 95% CI 1.059–1.263) compared to those under 20 years old, re-treated patients (aOR 1.143, 95% CI 1.066–1.226), and sputum smear-positive individuals (aOR 1.228, 95% CI 1.159–1.301), while waiters (aOR 0.869, 95% CI 0.8–0.943) and active case finding (aOR 0.767, 95% CI 0.737–0.798) act as protective factors (Table 3). Among migrant populations, waiters (aOR 0.836, 95% CI 0.782–0.893), retired staff (aOR 0.804, 95% CI 0.690–0.936), and active case finding (aOR 0.782, 95% CI 0.749–0.816) are protective factors, yet sputum smear-positive migrants have a notably higher diagnostic delay risk (aOR 1.349, 95% CI: 1.264–1.441) than local sputum smear-positive individuals (aOR 1.228, 95% CI: 1.159–1.301) (Table 4).

**Table 3:** Risk factors for patient, hospital and diagnosis delays identified by multivariable logistic regression among local PTB patients.

Characters (local)	Patient delay		Hospital delay		Diagnosis delay	
	aOR (95%CI)	*p*-value	aOR (95%CI)	*p*-value	aOR (95%CI)	*p*-value
Sex: female (vs. male)	**1.067 (1.018, 1.118)**	**0.007**	1.012 (0.967, 1.06)	0.606	**1.055 (1.012, 1.1)**	**0.012**
Age: (vs. ≦20 years)						
21–30 years	**1.213 (1.091, 1.347)**	**<0.001**	0.925 (0.843, 1.015)	0.101	1.045 (0.959, 1.139)	0.316
31–40 years	**1.439 (1.291, 1.605)**	**<0.001**	**0.888 (0.805, 0.98)**	**0.018**	**1.12 (1.024, 1.226)**	**0.014**
41–50 years	**1.588 (1.428, 1.766)**	**<0.001**	**0.87 (0.789, 0.958)**	**0.005**	**1.144 (1.047, 1.251)**	**0.003**
51–60 years	**1.635 (1.475, 1.813)**	**<0.001**	**0.867 (0.789, 0.952)**	**0.003**	**1.165 (1.069, 1.269)**	**0.001**
≧61 years	**1.609 (1.448, 1.787)**	**<0.001**	**0.878 (0.798, 0.967)**	**0.008**	**1.156 (1.059, 1.263)**	**0.001**
Ethic: minority (vs. Han)	0.89 (0.665, 1.19)	0.430	0.95 (0.722, 1.249)	0.712	0.918 (0.715, 1.177)	0.499
Occupation (vs. unemployment)						
Worker	0.968 (0.915, 1.024)	0.253	1.028 (0.972, 1.087)	0.329	1.023 (0.972, 1.076)	0.385
Official staff	**0.898 (0.812, 0.993)**	**0.035**	1.019 (0.925, 1.122)	0.706	0.952 (0.873, 1.039)	0.270
Waiter	**0.7 (0.634, 0.774)**	**<0.001**	**1.109 (1.015, 1.212)**	**0.022**	**0.869 (0.8, 0.943)**	**0.001**
Retired staff	**0.902 (0.841, 0.967)**	**0.003**	1.013 (0.944, 1.087)	0.722	0.963 (0.904, 1.026)	0.242
Case finding: active (vs. passive)	**0.881 (0.843, 0.921)**	**<0.001**	0.783 (0.75, 0.818)	<0.001	**0.767 (0.737, 0.798)**	**<0.001**
History: re-treated (vs. newly treated)	**1.101 (1.021, 1.186)**	**0.012**	**1.1 (1.018, 1.187)**	**0.015**	**1.143 (1.066, 1.226)**	**<0.001**
Smear-Positivity	**1.514 (1.419, 1.616)**	**<0.001**	**0.927 (0.87, 0.989)**	**0.021**	**1.228 (1.159, 1.301)**	**<0.001**
Bacteria Positivity	1.064 (0.997, 1.136)	0.063	0.96 (0.903, 1.022)	0.200	1.013 (0.958, 1.072)	0.643

aOR, adjusted Odds Ratio; CI, Confidential Interval.The bolded items indicate statistically significant results (*P* < 0.05).

**Table 4:** Risk factors for patient, hospital and diagnosis delays identified by multivariable logistic regression among migrant PTB patients.

Characters (migrant)	Patient delay		Hospital delay		Diagnosis delay	
	aOR (95%CI)	*p*-value	aOR (95%CI)	*p*-value	aOR (95%CI)	*p*-value
Sex: female (vs. male)	**1.159 (1.103, 1.218)**	**<0.001**	0.953 (0.908, 1.001)	0.057	**1.056 (1.01, 1.105)**	**0.017**
Age: (vs. ≦20 years)						
21–30 years	0.974 (0.884, 1.073)	0.593	0.942 (0.858, 1.033)	0.203	0.948 (0.87, 1.033)	0.222
31–40 years	1.087 (0.981, 1.204)	0.109	**0.902 (0.818, 0.995)**	**0.040**	0.979 (0.894, 1.072)	0.648
41–50 years	**1.146 (1.033, 1.273)**	**0.010**	0.932 (0.843, 1.031)	0.172	1.035 (0.943, 1.137)	0.464
51–60 years	**1.178 (1.054, 1.318)**	**0.004**	**0.875 (0.784, 0.976)**	**0.016**	1.007 (0.911, 1.113)	0.895
≧61 years	**1.278 (1.121, 1.457)**	**<0.001**	0.898 (0.788, 1.023)	0.106	1.077 (0.956, 1.214)	0.221
Ethic: minority (vs. Han)	**1.266 (1.121, 1.43)**	**<0.001**	0.892 (0.786, 1.012)	0.075	1.076 (0.96, 1.207)	0.210
Occupation (vs. unemployment)						
Worker	1.007 (0.949, 1.069)	0.814	1.011 (0.953, 1.071)	0.724	1.011 (0.958, 1.066)	0.702
Official staff	**0.84 (0.738, 0.955)**	**0.008**	1.001 (0.885, 1.131)	0.992	0.918 (0.821, 1.025)	0.128
Waiter	**0.746 (0.689, 0.807)**	**<0.001**	0.993 (0.923, 1.068)	0.845	**0.836 (0.782, 0.893)**	**<0.001**
Retired staff	**0.762 (0.643, 0.902)**	**0.002**	0.916 (0.771, 1.087)	0.316	**0.804 (0.69, 0.936)**	**0.005**
Case finding: active (vs. passive)	1.002 (0.955, 1.051)	0.946	**0.733 (0.7, 0.768)**	**<0.001**	**0.782 (0.749, 0.816)**	**<0.001**
History: re-treated (vs. newly treated)	1.094 (0.986, 1.215)	0.090	1.045 (0.938, 1.164)	0.424	**1.118 (1.013, 1.234)**	**0.027**
Smear-Positivity	**1.823 (1.694, 1.963)**	**<0.001**	**0.88 (0.819, 0.945)**	**<0.001**	**1.349 (1.264, 1.441)**	**<0.001**
Bacteria Positivity	1.013 (0.943, 1.089)	0.715	1.007 (0.942, 1.076)	0.845	1.01 (0.95, 1.074)	0.749

aOR, adjusted Odds Ratio; CI, Confidential Interval.The bolded items indicate statistically significant results (*P* < 0.05).

For patient delay, female sex was a significant risk factor in both groups, with a higher effect size among migrants (aOR 1.159, 95% CI: 1.103–1.218) compared with local residents (aOR 1.067, 95% CI: 1.018–1.118). Ethnic minority status was associated with increased patient delay only in migrants (aOR 1.266, 95% CI: 1.121–1.430), whereas no significant association was observed in local residents (aOR 0.890, 95% CI: 0.665–1.190). Sputum smear-positivity conferred a more pronounced risk in migrants (aOR 1.823, 95% CI: 1.694–1.963) compared with local residents (aOR 1.514, 95% CI: 1.419–1.616). Retired patients exhibited lower patient delay risk in both groups, with a stronger protective effect among migrants (aOR 0.762, 95% CI: 0.643–0.902) than local residents (aOR 0.902, 95% CI: 0.841–0.967). In contrast to patient and diagnostic delays, the magnitude of differences in factor effects between migrants and local residents was relatively small for hospital delay.

## 4 Discussion

The global pandemic was still ongoing, and the situation of epidemic prevention and control was still severe. The long-term epidemiological trend of PTB deserved continuous attention (22, 23). Our government always attached great importance to PTB prevention and treatment. Since 2001, the State Council issued three National Tuberculosis Prevention and Control Programs and a series of programmatic documents on PTB prevention and control (24–27). This study focuses on the trends and risk factors for patient, hospital and diagnosis delays among migrant TB patients in Guangzhou from 2014 to 2022. During the study period, the number of TB patients in Guangzhou decreased annually, with a corresponding decline in the reported incidence rate.

A total of 35,722 migrant PTB patients were mainly characterized by a higher percentage of females (34.14% vs. 30.69%), a greater proportion of ethnic minorities (3.49% vs. 0.57%), a higher rate of newly diagnosed patients (95.08% vs. 91.78%), and a younger age structure, with a significantly higher proportion of individuals under 40 years (64.02% vs. 36.46%). The occupations of these migrants were mostly blue-collar workers in the service industry, workers, and unemployed individuals with generally lower incomes, potentially facing greater healthcare barriers such as inconvenient medical insurance reimbursement, limited time, and insufficient social support (28), which may increase their risk of delayed diagnosis.

During the study period, 27.90% of patients experienced patient delay >30 days, 29.09% had hospital diagnostic delay >14 days, and 44.39% had diagnostic delay >30 days. Although the overall delay rates for both local and migrant TB patients in Guangzhou showed a downward trend from 2014 to 2022, multivariate logistic regression indicated that migrants had a significantly higher risk of delays. Key risk factors for patient and diagnostic delays among migrants included female sex, older age, and ethnic minority status. These findings align with a meta-analysis (29) involving 19 low-and lower-middle-income countries, where females, ethnic minorities, and older patients often exhibit lower healthcare awareness and weaker willingness to seek medical services (30). In fact, these groups are also more likely to face substantial economic burdens related to TB treatment costs (31, 32), highlighting the need for targeted TB prevention and control efforts for these high-risk populations.

Sputum smear-positivity was identified as a risk factor for both patient and diagnostic delays but a protective factor for hospital diagnosis. As shown in Figure 2, patients with negative bacteriological results had relatively higher hospital delay rates, consistent with a study in Ethiopia (33). Bacteriologically negative or sputum smear-negative patients often await culture results and subsequent medical consultations, a process that prolongs hospital delays. However, sputum smear-positive patients, due to their high infectivity from abundant *Mycobacterium tuberculosis* in sputum, pose an increased risk of disease transmission when experiencing care-seeking delays, threatening public health security. Moreover, active case-finding was a protective factor against care-seeking delays, yet passive case finding accounted for 46.15% of cases, possibly due to non-specific TB symptoms (prone to misdiagnosis with other diseases) and patients’ fears of social stigma and isolation following a positive diagnosis. This underscores the urgency of strengthening health education to encourage earlier and more proactive screening (34).

Bacteriological examination is a primary step in identifying infectious sources and effectively controlling TB epidemics. Rapid and accurate bacteriological testing is critical to reducing misdiagnosis and enabling effective TB diagnosis and treatment. Our data showed that the bacteriological positivity rate among migrants was lower than that among local residents, possibly due to: (1) a higher proportion of newly treated migrant patients with lower sputum test compliance, reducing detected positive cases; (2) potential quality issues with sputum specimens, especially among newly treated migrants. Studies have shown that interventions to improve sputum specimen quality can significantly increase bacteriological positivity in newly diagnosed patients (28), and (3) limited economic and medical insurance support, leading to lower utilization of molecular testing among migrants. To address these issues, efforts should be made to enhance the living conditions and lifestyle of the migrants, increase the rate of sputum examination among newly treated patients, elevate the quality of sputum specimens, and provide financial and health insurance support.

The limitation of this study is that self-reported symptom onset dates may be subjective, potentially introducing reporting bias. While the logistic regression model identified risk factors for TB diagnostic delay, it has limitations in inferring causal relationships, warranting further research.

In conclusion, our study reveals the trends and risk factors for patient, hospital and diagnosis delays among PTB patients in Guangzhou from 2014 to 2022. These findings are valuable for formulating targeted TB prevention and control policies and interventions for migrant populations.

## Data Availability

The original contributions presented in the study are included in the article/Supplementary material, further inquiries can be directed to the corresponding authors.
