# Supplementary material for: Analysis of diagnosis delay among migrant pulmonary tuberculosis patients in Guangzhou from 2014 to 2022
Source: Front Public Health. 2025 May 14;13:1399688. doi: 10.3389/fpubh.2025.1399688 (PMC12116643; doi:10.3389/fpubh.2025.1399688)
Supplement: Supplementary file 1 [file Supplementary_file_1.docx]

Supplementary Table 1 Between-group comparison of factors related to patient delay.

| **Characters** | **Non-patient delay** | **Patient delay** | **χ^2^** | ***P*-value** |
| --- | --- | --- | --- | --- |
| Gender |  |  | 2.33 | 0.127 |
| male | 39,120 (72.27%) | 18,474 (71.75%) |  |  |
| female | 15,010(27.73%) | 7,273 (28.25%) |  |  |
| Age |  |  | 258.67 | <0.001 |
| ≦20 | 4,326（7.51%） | 1,310（5.88%） |  |  |
| 21-30 | 15,258（26.49%） | 5,044（22.64%） |  |  |
| 31-40 | 9,432（16.38%） | 3,598（16.15%） |  |  |
| 41-50 | 8,794（15.27%） | 3,746 (16.81%) |  |  |
| 51-60 | 8,639（15.00%） | 3,775（16.94%） |  |  |
| ≧61 | 11,145（19.35%） | 4,810（21.59%） |  |  |
| Ethnicity |  |  | 14.34 | <0.001 |
| han | 56,579 (72.19%) | 21,800 (27.81%) |  |  |
| minority | 1,015 (67.76%) | 483 (32.24%) |  |  |
| Census register |  |  | 3.92 | 0.048 |
| local | 31,962 (55.50%) | 12,193 (54.72%) |  |  |
| migrant | 25,632 (44.50%) | 10,090 (45.28%) |  |  |
| Occupation |  |  | 172.68 | <0.001 |
| unemployment | 30,612 (53.15%) | 12,087 (54.24%) |  |  |
| worker | 12,075 (20.97%) | 5,077 (22.78%) |  |  |
| official staff | 2,805 (4.87%) | 929 (4.17%) |  |  |
| waiter | 5,497 (9.54%) | 1,543 (6.92%) |  |  |
| retired staff | 6,605 (11.47%) | 2,647 (11.88%) |  |  |
| Case detection |  |  | 39.56 | <0.001 |
| active | 26,182 (45.46%) | 10,681 (47.93%) |  |  |
| passive | 31,412 (54.54%) | 11,602 (52.07%) |  |  |
| Treatment history |  |  | 82.03 | <0.001 |
| newly treated | 53,997 (93.75%) | 20,492 (91.96%) |  |  |
| re-treated | 3,597 (6.25%) | 1,791 (8.04%) |  |  |
| First sputum smear status |  |  | 1,202.18 | <0.001 |
| negative | 39,319 (68.27%) | 12,298 (55.19%) |  |  |
| positive | 18,275 (31.73%) | 9,985 (44.81%) |  |  |
| Bacteriological results |  |  | 707.93 | <0.001 |
| negative | 30,312 (52.63%) | 9,389 (42.14%) |  |  |
| positive | 27,282 (47.37%) | 12,894 (57.86%) |  |  |

Supplementary Table 2 Between-group comparison of factors related to hospital delay.

| **Characters** | **Non-hospital delay** | **Hospital delay** | **χ^2^** | ***P*-value** |
| --- | --- | --- | --- | --- |
| Gender |  |  | 0.87 | 0.350 |
| male | 38,327 (67.67%) | 15,803 (68.01%) |  |  |
| female | 18,313 (32.33%) | 7,434 (31.99%) |  |  |
| Age |  |  | 28.50 | <0.001 |
| ≦20 | 3,897 (6.88%) | 1,739 (7.48%) |  |  |
| 21-30 | 14,237 (25.14%) | 6,065 (26.10%) |  |  |
| 31-40 | 9,230 (16.30%) | 3,800 (16.35%) |  |  |
| 41-50 | 8,876 (15.67%) | 3,664 (15.77%) |  |  |
| 51-60 | 8,917 (15.74%) | 3,497 (15.05%) |  |  |
| ≧61 | 11,483 (20.27%) | 4,472 (19.25%) |  |  |
| Ethnicity |  |  | 0.63 | 0.429 |
| han | 55,564 (98.10%) | 22,815 (98.18%) |  |  |
| minority | 1,076 (1.90%) | 422 (1.82%) |  |  |
| Census register |  |  | 5.39 | 0.020 |
| local | 31,458 (55.54%) | 12,697 (54.64%) |  |  |
| migrant | 25,182 (44.46%) | 10,540 (45.36%) |  |  |
| Occupation |  |  | 26.50 | <0.001 |
| unemployment | 30,299 (53.49%) | 12,400 (53.36%) |  |  |
| worker | 11,992 (21.17%) | 5,160 (22.21%) |  |  |
| official staff | 2,681 (4.73%) | 1,053 (4.53%) |  |  |
| waiter | 4,939 (8.72%) | 2,101 (9.04%) |  |  |
| retired staff | 6,729 (11.88%) | 2,523 (10.86%) |  |  |
| Case detection |  |  | 301.54 | <0.001 |
| active | 25,028 (44.19%) | 11,835 (50.93%) |  |  |
| passive | 31,612 (55.81%) | 11,402 (49.07%) |  |  |
| Treatment history |  |  | 0.01 | 0.990 |
| newly treated | 52,819 (93.25%) | 21,670 (93.26%) |  |  |
| re-treated | 3,821 (6.75%) | 1,567 (6.74%) |  |  |
| First sputum smear status |  |  | 39.37 | <0.001 |
| negative | 36,216 (63.94%) | 15,401 (66.28%) |  |  |
| positive | 20,424 (36.06%) | 7,836 (33.72%) |  |  |
| Bacteriological results |  |  | 34.98 | <0.001 |
| negative | 27,772 (49.03%) | 11,929 (51.34%) |  |  |
| positive | 28,868 (50.97%) | 11,308 (48.66%) |  |  |

Supplementary Table 3 Between-group comparison of factors related to diagnosis delay.

| **Characters** | **Non-diagnosis delay** | **Diagnosis delay** | **χ^2^** | ***P*-value** |
| --- | --- | --- | --- | --- |
| Gender |  |  | 0.312 | 0.577 |
| male | 26,453（67.86%） | 27,677（67.68%） |  |  |
| female | 12,528（32.14%） | 13,219（32.32%） |  |  |
| Age |  |  | 45.93 | <0.001 |
| ≦20 | 2,861（7.34%） | 2,775（6.79%） |  |  |
| 21-30 | 10,212（26.20%） | 10,090（24.67%） |  |  |
| 31-40 | 6,356（16.31%） | 6,674（16.32%） |  |  |
| 41-50 | 5,929（15.21%） | 6,611（16.17%） |  |  |
| 51-60 | 5,933（15.22%） | 6,481（15.85%） |  |  |
| ≧61 | 7,690（19.73%） | 8,265（20.21%） |  |  |
| Ethnicity |  |  | 4.81 | 0.028 |
| han | 38,292（98.23%） | 40,087（98.02%） |  |  |
| minority | 689（1.77%） | 809（1.98%） |  |  |
| Census register |  |  | 12.75 | <0.001 |
| local | 21,799（55.92%） | 22,356（54.67%） |  |  |
| migrant | 17,182（44.08%） | 18,540（45.33%） |  |  |
| Occupation |  |  | 106.43 | <0.001 |
| unemployment | 20,740（53.21%） | 21,959（53.69%） |  |  |
| worker | 7,961（20.42%） | 9,191（22.47%） |  |  |
| official staff | 1,933（4.96%） | 1,801（4.40%） |  |  |
| waiter | 3,728（9.56%） | 3,312（8.10%） |  |  |
| retired staff | 4,619（11.85%） | 4,633（11.33%） |  |  |
| Case detection |  |  | 369.42 | <0.001 |
| active | 16,636（42.68%） | 20,227（49.46%） |  |  |
| passive | 22,345（57.32%） | 20,669（50.54%） |  |  |
| Treatment history |  |  | 39.76 | <0.001 |
| newly treated | 36,575（93.83%） | 37,914（92.71%） |  |  |
| re-treated | 2,406（6.17%） | 2,982（7.29%） |  |  |
| First sputum smear status |  |  | 358.65 | <0.001 |
| negative | 26,469（67.90%） | 25,148（61.49%） |  |  |
| positive | 12,512（32.10%） | 15,748（38.51%） |  |  |
| Bacteriological results |  |  | 187.58 | <0.001 |
| negative | 20,342（52.18%） | 19,359（47.34%） |  |  |
| positive | 18,639（47.82%） | 21,537（52.66%） |  |  |
